# Genetic and Environmental Influences on Singing Self-Evaluation and its Relationship with Singing Ability: An Australian Twin Study

**DOI:** 10.1007/s10519-026-10254-4

**Published:** 2026-02-09

**Authors:** Daniel Yeom, Kendall S. Stead, Yi Ting Tan, Gary E. McPherson, Miriam A. Mosing, Sarah J. Wilson

**Affiliations:** 1 Melbourne School of Psychological Sciences, University of Melbourne, Melbourne, VIC 3010 Australia; 2 School of Psychological Sciences, Macquarie University, Sydney, NSW 2109 Australia; 3 Melbourne Conservatorium of Music, University of Melbourne, Southbank, VIC 3006 Australia; 4 Department of Neuroscience, Karolinska Institutet, 17177 Stockholm, Sweden; 5 Department of Medical Epidemiology and Biostatistics, Karolinska Institutet, 17177 Stockholm, Sweden; 6 Department of Medicine, Epilepsy Research Centre, University of Melbourne, Austin Health, Heidelberg, VIC 3084 Australia

**Keywords:** Singing ability, Singing self-evaluation, Heritability, Music genetics

## Abstract

People evaluate their singing ability to varying degrees of accuracy, but why these individual differences occur is largely unknown. Objectively evaluated, everyday singing ability is influenced by both genetic and environmental factors. Since singing self-evaluation (SSE) strongly correlates with everyday singing ability, it is possible that the same factors might influence both. Using a classical twin design, we estimated genetic and environmental influences on SSE and its phenotypic relationship with everyday singing ability in Australian twins (*N* = 996). Two validated measures of SSE were used: a single self-report item assessing overall singing ability (SSE-Single) and a four-item composite measure (SSE-Factor). Both measures showed significant genetic (SSE-Single *A* = 42%; SSE-Factor *A* = 53%) and unshared environmental influences (both *E* = 29%). Phenotypic correlations between the SSE measures and everyday singing ability ranged from *r*_*p*_ = 0.67 − 0.68 and were significantly influenced by genetic, shared and unshared environmental factors (SSE-Single *A* = 41%, *C* = 51%, *E* = 8%; SSE-Factor *A* = 37%, *C* = 52%, *E* = 11%). Strong genetic correlations (*r*_*g*_ = 0.63 − 0.64) were observed between SSE measures and everyday singing ability, as well as between both SSE measures (*r*_*g*_ = 0.82), suggesting shared underlying genes. Taken together, our findings indicate that both genes and environments affect how individuals evaluate their own singing ability and how accurate these evaluations are compared to their actual ability.

**Supplementary Information:**

The online version contains supplementary material available at 10.1007/s10519-026-10254-4.

## Introduction

Singing is universal across cultures (Mehr et al. 2019) and facilitates prosocial behaviour and social bonding (Clift et al. 2010; Pearce et al. 2016; Trehub et al. 2015; Weinstein et al. 2016). There is consistent evidence that singing fosters biological, psychological and social benefits (e.g., Daykin et al. 2018; Reagon et al. 2016; Stacy et al. 2002). Since singing is an innately developing ability with wide natural variation (Yeom et al. 2022), it is a valuable phenotype for studying genetic and environmental influences on the development of beneficial human traits. Importantly, people also hold strong but varied self-concepts about their own singing abilities. These self-concepts can have powerful effects on the ways in which people engage with singing activities and subsequently experience its benefits. Thus, the relationship between, and determinants of, variability in objectively evaluated everyday singing ability and self-evaluation warrant further investigation, offering insights into the biological, psychological and social processes that shape musical engagement and its benefits.

### Subjective Evaluation of Objective Skills

Individuals are generally accurate when assessing their current state, such as health status with self-rated health reliably correlating with key health predictors (Eriksson et al. 2001; Wu et al. 2013). However, they are less accurate when asked to evaluate their own abilities or skills. In a meta-synthesis Zell and Krizan (2014) reported an average correlation of *r* = 0.29 between self-rated and objective abilities in domains including sports, academic and cognitive abilities.

Despite this, previous research has demonstrated a relationship between how individuals evaluate their singing ability (referred to as *singing self-evaluation*,* SSE*) and their actual ability, with correlations ranging from *r* = 0.18–0.41 and *ρ* = 0.44–0.49 (Pfordresher 2022; Pfordresher and Demorest 2021). While these correlations indicate that individuals are reasonably accurate in rating their singing ability, misestimation of ability is also common. For example, some individuals demonstrate high singing proficiency despite low self-evaluations, while others evaluate themselves highly but perform poorly (Larrouy-Maestri et al. 2023; Pfordresher and Demorest 2021). This misestimation may be attributed to a combination of motivational, psychological and methodological factors, including the perceived difficulty of tasks, task familiarity, and the use of objective measures compared to expert or peer ratings of performance (Zell and Krizan 2014).

The accuracy of people’s self-evaluations has crucial ramifications for minimal phenotyping. Minimal phenotyping typically involves the use of simple and brief self-rating measures to measure a trait, as opposed to more comprehensive data collection methods such as objective test batteries (i.e., deep phenotyping). Minimal phenotypic measures are increasingly used in genome wide association studies (GWAS) and similar population-level genomic methods to advance gene discovery, as they reduce costs and capture larger samples efficiently. However, this approach assumes that minimal measures accurately index the phenotype of interest. If a measure fails to capture a phenotype accurately – as is the case when people strongly misestimate their own abilities – genetic loci not specific to the trait may be overidentified (Cai et al. 2020). This can bias our understanding of the trait’s genetic architecture and the application of genetic findings. In addition, there is a trade-off between efficiency and comprehensiveness between different minimal phenotypic measures (Jamshidi et al. 2020). While factorial approaches based on multiple items typically capture the complexity of a trait more comprehensively, single items are easier and more cost-efficient to disseminate. Thus, it is critical to test the validity of various minimal phenotypic measures to determine how accurately they capture phenotypes.

### Phenotyping Singing with the Melbourne Singing Tool

We have previously developed a well-validated online tool, the Melbourne Singing Tool (MST). This tool provides a deep phenotypic measure of singing ability, the Singing Phenotypic Index (SPI), based on multiple objective measures of everyday singing (Tan et al. 2021; Yeom et al. 2022). The MST also includes a self-report questionnaire (MST-Q) that generates two useful minimal phenotypic measures of SSE, namely the SSE-Single and SSE-Factor. SSE-Single comprises a single, self-report item, *“How much singing ability do you have?”*, whereas SSE-Factor comprises four self-report items explained by one latent factor (Yeom et al. 2023). While the SSE-Factor comprises of four items that relate to specific aspects of singing ability (e.g., “*After hearing a new song two or three times*,* I can usually sing/hum/whistle it by myself.”*), the SSE-Single is a separate item intended to measure a global, holistic self-evaluation of ability. Both measures show a robust correlation with the SPI (both *r* = 0.66) that is stronger than other MST-Q factors, including an individual’s social (*r* = 0.46) or personal (*r* = 0.36) engagement with singing and music correlate with the SPI. Notably, both SSE-Single and SSE-Factor predict the SPI to the same degree, highlighting the utility of the MST in providing comprehensive phenotypic measures that cater to both deep and minimal phenotyping approaches, facilitating more robust and accurate genetic studies.

Compared to what is typically expected from prior research, we have observed stronger correlations between self-evaluated and actual singing ability. To explain this discrepancy, we speculated that the MST’s objective tasks capture everyday singing behaviours that individuals often engage in, namely singing familiar tunes and learning new tunes. In turn, individuals may draw on their experiences from repeated singing attempts to inform their evaluations, in addition to other sources (Yeom et al. 2023). This is consistent with accounts in non-singing domains where people integrate feedback from various sources to evaluate their abilities. Individuals typically receive feedback from others and compare their own abilities to others or their past selves, thereby reinforcing existing self-conceptions (Taylor et al. 1995; Wilson and Ross 2000).

### Genetic and Environmental Influences on Self-evaluations

Twin designs provide a powerful way of unpacking genetic influences on self-evaluated and objectively evaluated abilities, and their relationship. The classical twin design is used in behavioural genetics to estimate the genetic and environmental influences of traits by comparing similarities between monozygotic (MZ) or identical twins, and dizygotic (DZ) or fraternal twins (Boomsma et al. 2002). While MZ twins share 100% of their DNA, DZ twins share 50% on average; this twin similarity is leveraged to delineate genetic, shared environmental and unshared environmental influences on a trait’s variation. The method relies on the assumption that the twin pairs are reared in the same household and experience similar environments.

Research suggests that objective singing ability is moderately heritable (≈ 41%; Park et al. 2012; Yeom et al. 2022) and additionally influenced by both family environments (37.1%) and non-shared environmental factors (Yeom et al. 2022). However, to our knowledge research has not yet examined the genetic and environmental influences on self-evaluated singing ability, nor on the relationship it has with objective singing ability. In the absence of such research, twin studies examining academic self-evaluation provide valuable context. Previous findings largely suggest that genetics and non-shared environmental influences shape how individuals evaluate their academic abilities (Gottschling et al. 2012; Greven et al. 2009; Luo et al. 2011; Spinath et al. 2008; Starr and Riemann 2022). Given the well-replicated finding that most, if not all, psychological traits are heritable (Plomin et al. 2016; Polderman et al. 2015), the substantial contribution of genetic effects to self-evaluation in an academic context is not surprising.

Similar findings are observed in multivariate investigations of the phenotypic relationship between academic self-evaluation and academic performance. The contribution of genetic factors to a phenotypic correlation indicates the degree to which genes explain the association between traits at a phenotypic level (de Vries et al. 2021). Genetic influences have been identified in domains such as mathematics (Luo et al. 2011; Starr and Riemann 2022) and German language proficiency (Starr and Riemann 2022), while unshared environmental factors play a smaller role. Additionally, significant genetic correlations between academic self-evaluation and academic performance have been consistently reported (*r*_*g*_ = 0.59 − 0.79; Greven et al. 2009; Gottschling et al. 2012; Starr and Riemann 2022). Genetic correlations indicate the extent to which two phenotypes share underlying genetic variants, pointing to pleiotropy (de Vries et al. 2021). In turn, significant genetic correlations between self-evaluated and objective abilities may indicate that the self-evaluative measures capture meaningful genetic signal in an objective measure, thereby pointing to their potential viability as phenotypic measures in genetic studies. While research on academic self-evaluation highlights that both genetic and environmental factors underlie how individuals evaluate their own ability, whether they also directly apply to singing is unclear.

### The Present Study

The primary aim of this study was to examine the nature of the relationship between self-evaluated singing ability and objective, everyday singing ability, using the minimal and deep phenotypic measures of the MST in a large sample of Australian twins. Although previous literature largely suggests that genetic and non-shared environmental factors influence self-evaluations, our previous work shows that singing ability is unexpectedly shaped by both genetic and shared environmental influences (Yeom et al. 2022). Thus, we expected that the genetic and environmental factors that likely contribute to mechanisms for accurate singing (e.g. sensorimotor and other cognitive processes) might also be relevant for self-evaluation. To investigate this aim, we fit a multivariate twin model to test whether the same genetic factors influence both the subjective and objective evaluation of singing ability. We hypothesised that individual differences in SSE-Single and SSE-Factor would be substantially influenced by both genetic and shared environmental factors to a similar degree, as is the case for objectively assessed singing ability. We then explored genetic and environmental influences on the relationship of SSE-Single and SSE-Factor with the SPI.

## Methods

### Participants

We used the same sample of 996 participants from Yeom et al. (2023). Participants were recruited across Australia with the assistance of Twins Research Australia, as part of a study investigating the genetic basis of singing ability. Ethical approval for this study was given by the Human Research Ethics Committee of the University of Melbourne (ID 1750061). Of the original sample of 1,273 twins, 996 had complete objective singing and questionnaire data and were therefore included in the present analyses. Participants with missing data on either measure were excluded.

The final sample comprised 335 MZ (252 female, 83 male) and 118 DZ (85 female, 26 male, 7 opposite-sex) complete twin pairs. Singletons (*n* = 90; 33 MZ female, 20 DZ female, 15 MZ male and 22 MZ female) were retained in the initial set of analyses as they help estimate variances and covariances. The average age was 45.70 (SD = 16.27; range = 15–90) in this subsample. We winsorized self-reported years of music training to a maximum of 20 years to account for implausible values. Participants had 4.55 years of training on average (SD = 5.16; range = 0–20).

### Materials and Procedure

All participants completed the MST as described by Tan et al. (2021) and the MST-Q, as detailed by Yeom et al. (2023). In brief, the MST contains three singing tasks to objectively measure pitch accuracy of singing, while the MST-Q is a 16-item self-report questionnaire. Participants also completed the SSE-Single, providing a global evaluation of their singing ability (rated on a scale of 1 to 7).

#### Melbourne Singing Tool

The three singing tasks were designed to capture objective everyday singing ability in the general population. These tasks include:

1. Sing the Note task: Participants listen to a series of individual notes and sing them back as accurately as possible. The frequency range of the note differed for females and males.
2. Familiar Tune task: Participants sing ‘Happy Birthday’ to a paced drum track. Three trials are recorded for this task. Participants sing with lyrics on the first trial, and then on the syllable “dah” on subsequent trials. Participants can start on a note of their choice, allowing them to sing in a comfortable range.
3. Unfamiliar Tune task: Participants listen to seven-note melodies and sing them back as accurately as possible. The frequency range of the notes in the melodies differ for females and males.

Five measures of singing accuracy were extracted from the three tasks, using the open-source *TONY* software (Mauch et al. 2015). Across all tasks, the average pitch deviation was calculated to reflect the average absolute difference between each sung note and target note, across all trials. For the Familiar and Unfamiliar Tune tasks, we also calculated average interval deviation, which reflects the absolute difference between each sung *interval* (i.e. the distance between two adjacent notes) and the target interval. All five measures were in cents, where 100 cents equals a semitone. We have previously shown that these five accuracy measures load strongly onto one latent factor representing singing ability. We derived a factor score from this latent factor termed the Singing Phenotypic Index (SPI; Yeom et al. 2022), which serves as a deep, objective phenotypic measure of singing ability. Further information on how these measures were extracted and calculated are available in Tan et al. (2021) and Yeom et al. (2022).

#### Melbourne Singing Tool Questionnaire (MST-Q)

The MST-Q is a brief 16-item questionnaire designed to measure engagement with singing in different contexts and evaluate specific aspects of singing ability. All 16 items are rated on a 5-point Likert scale (*1 = Never*,* 5 = Always*). In a previous study (Yeom et al. 2023), we used exploratory factor analysis to show that three latent factors underlie responses to the 16 items:

4. Personal Engagement (six items), representing singing engagement in individual contexts,
5. Social Engagement (six items), representing engagement in contexts involving others, such as family and friends, and.
6. Self-Evaluation of Singing (SSE-Factor, four items), capturing all items that required participants to evaluate themselves.

Factor scores for each of the three latent factors were calculated using the Thurstone regression method (Thurstone 1935), which derives individual scores from the factor loadings. In addition to the MST-Q, participants completed a singing self-evaluation single-item (SSE-Single), a single 7-point item asking them to globally evaluate their singing ability (*How much singing ability do you have? 1 = Not at all*,* 7 = A great deal*).

As reported in our previous study (Yeom et al. 2023), the MST-Q demonstrates high internal consistency (McDonald’s ω_t_ = 0.92; Cronbach’s α = 0.90, 95% CI: [0.89, 0.91]). The three MST-Q factors and the SSE-Single significantly and positively correlate with the objective SPI to varying degrees, indicating good construct validity. Specifically, the SSE-Factor and SSE-Single had the strongest relationships to singing ability (*r* = 0.66), followed by Social Engagement (*r* = 0.46) and Personal Engagement (*r* = 0.36). Both singing self-evaluation measures were also shown to predict the SPI to the same degree ($\:\beta\:$ = 0.386; Yeom et al. 2023). See Yeom et al. (2023) for further details on these validity and reliability measures. Our primary focus is on these singing self-evaluation measures as minimal phenotypic measures of singing ability. The Personal and Social Engagement factors were not analysed in the present study as we were specifically interested in self-evaluation of singing.

### Data Analyses

All analyses were conducted in R version 4.4.2 (R Core Team 2023) and OpenMx version 2.21.13 (Boker et al. 2011; Neale et al. 2016), with the NPSOL estimator. We ran assumption tests to assess equalities in means, variances and covariances between twin order, zygosities and sexes for each variable. We first fit a saturated model that estimated all means, variances and covariances, then fit nested submodels that constrained parameters. Means were first constrained to be equal across twin order, zygosity and sex, then variances were constrained in the same order. Covariances were constrained across sexes and zygosities. The fit of each submodel was compared to the previous nested submodel using likelihood ratio tests, where the minus 2 log likelihood (−2LL) of each model was compared to assess changes in model fit. For each comparison, we set a significance level of α = 0.01. Significant differences in −2LL indicate worse model fit. No significant differences for parameters between subgroups were found (Table S1).

To address our main hypothesis we fit a series of univariate and multivariate twin models with sex and age as covariates in all models. We first fit three univariate models for the SSE-Factor, the SSE-Single, and the SPI. Intraclass twin correlations for MZ and DZ twins were estimated for each variable. The ratio of twin correlations between MZ and DZ twins determines whether to fit a univariate ACE or ADE model, as shared environmental influences (C) and dominant genetic effects (D) are confounded in a classical twin design. If the MZ twin correlation is less than twice as high as the DZ correlations (*r*_MZ_ < 2*r*_DZ_), an ACE model is indicated.

**Fig. 1:**
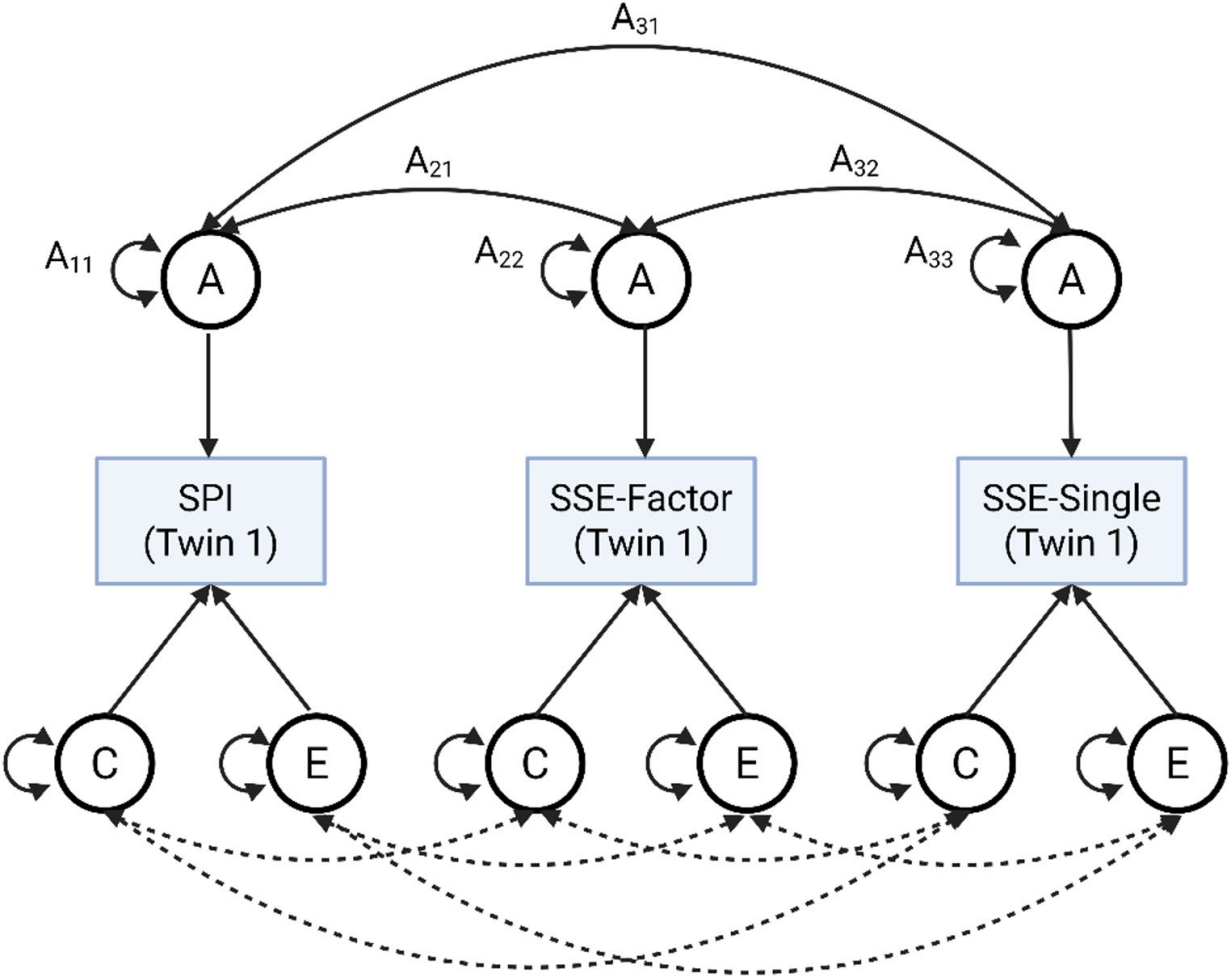
Conceptual diagram of the multivariate correlated factors model. Each A with subscripts (e.g. A_11_) indicates a parameter (variance or covariance) estimated in the model. Genetic/environmental components are denoted by circles. Double-headed arrows on a component indicate variances; double-headed arrows between components indicate covariances. For visualisation purposes, parameter labels have only been added on the parameters for additive genetic (A) effects. However, note that the same apply to shared and unshared environmental variances/covariances as well, as indicated by the dashed lines. In addition, only twin 1 is shown for visualisation purposes. *A* = additive genetic effects; *C* = shared environmental effects; *E* = unshared environmental effects; *SPI *= Singing Phenotypic Index; *SSE-Factor* = Singing Self-Evaluation Factor (4 items); *SSE-Single* = Singing Self-Evaluation Single Item. Graphic created in BioRender.

Next, to explore the relationship between self-evaluated and objective singing ability we fit a multivariate correlated factors model on the SSE-Single, SSE-Factor and the SPI (Fig. 1). We used direct symmetric parameterisation to estimate A, C and E variance components on each variable (Verhulst et al. 2019), as well as pairwise genetic and environmental correlations between the ACE components (e.g. see Bignardi et al. 2025). In turn, this model allows for estimation of the genetic and environmental contributions to the phenotypic correlation of each relationship. We chose this model because as per our aims, we were mainly interested in examining genetic and environmental correlations between the self-evaluative and objective measures. Although alternative models exist (e.g. common pathway, independent pathway models), both models imply a shared genetic factor common to all variables (Rijsdijk and Sham 2002). However, as we view the self-evaluated measures as conceptually distinct from the objective measure, the correlated factors model provides a more conceptually plausible model for our research questions. We also tested whether more restricted AE, CE and E models fit better than the full ACE using likelihood ratio tests. For completeness, we report the full ACE results for both the univariate and multivariate analyses regardless of significance.

Genetic correlations were calculated using the following formula (de Vries et al. 2021; Rijsdijk and Sham 2002):

$$\:{r}_{g}=\:\frac{{A}_{21}}{\sqrt{{A}_{11}\:\times\:\:{A}_{22}}}$$

Where A_11_ and A_22_ represent the genetic variances on traits 1 and 2 respectively, and A_21_ represents the genetic covariance between the two traits. The contribution of genetic factors to the phenotypic correlation can then be calculated using the following formula:

$$\:Bivariate\:A=\:\frac{\sqrt{{A}_{11}}\:\times\:\:{r}_{g}\:\times\:\:\sqrt{{A}_{22}}}{{r}_{p}}$$

Where A_11_ and A_22_ represent the genetic variances on each trait, *r*_*g*_ is the genetic correlation between the two traits and *r*_*P*_ represents the phenotypic correlation. These formulae can also be used to calculate shared and unshared environmental correlations (*r*_*c*_ and *r*_*e*_), and in turn, the contribution of C and E to the phenotypic correlation by substituting C and E variance components respectively into both equations.

## Results

### Univariate Analyses

**Fig. 2:**
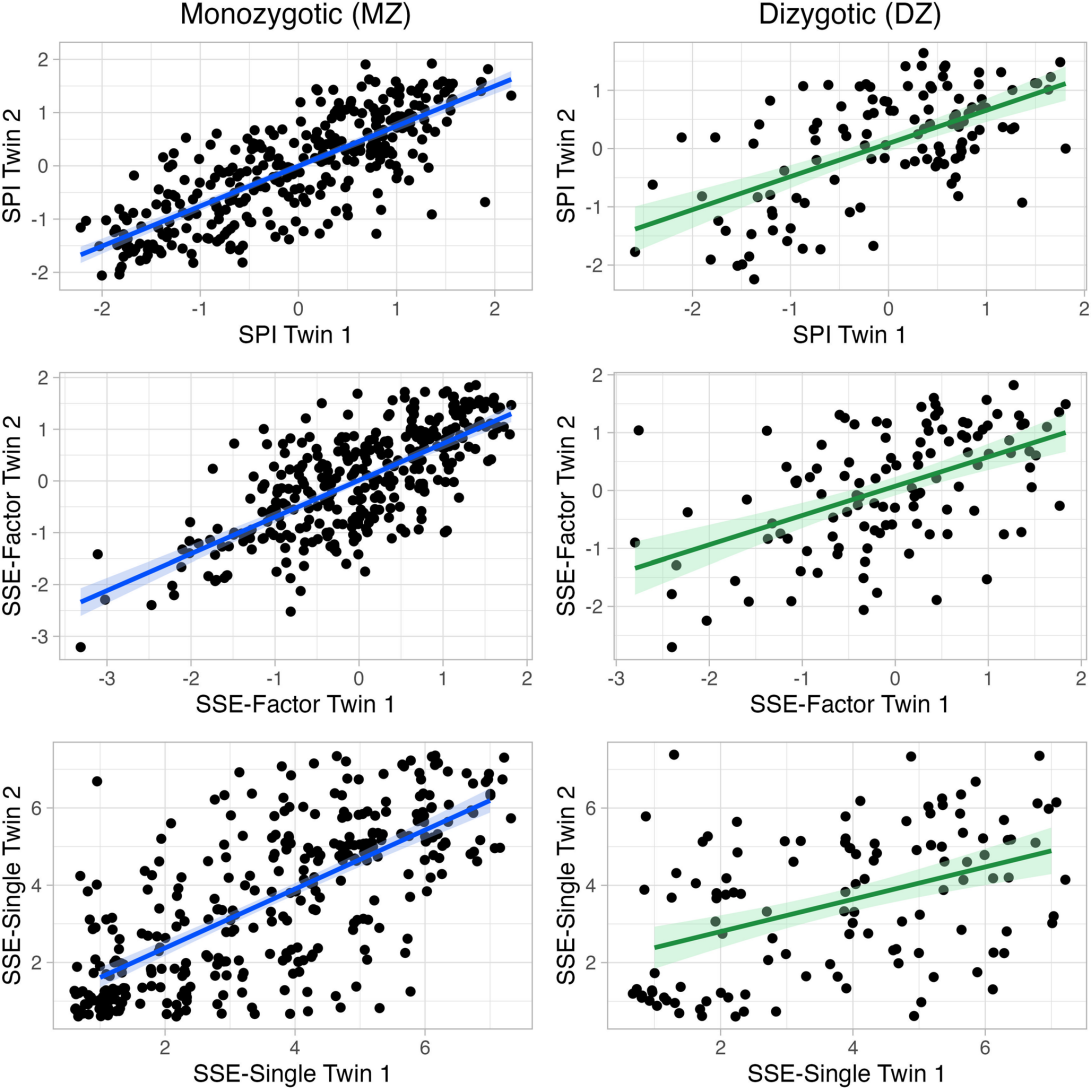
Intraclass twin correlations for monozygotic (MZ) and dizygotic (DZ) twins. *SPI* = singing phenotypic index, *SSE-Factor* = singing self-evaluation factor, *SSE-Single* = singing self-evaluation single item. SSE-Single data points were jittered to better visualise discrete values. Shaded areas around the line represent the 95% confidence interval.

DZ intraclass twin correlations were more than half of the corresponding MZ correlations for the SPI (MZ = 0.77, DZ = 0.60), SSE-Factor (MZ = 0.70, DZ = 0.52) and SSE-Single (MZ = 0.71, DZ = 0.44), indicating ACE models for all univariate analyses (Fig. 2). Results from the three univariate analyses are presented in Table 1 and model fitting statistics for the full and nested model comparisons are presented in Table S2. The univariate model for the SPI yielded similar results (A = 35.2%, C = 41.9%, E = 22.9%) to our previous paper (Yeom et al. 2022), with any differences owed to including only twins with complete MST-Q data. Additive genetic effects were significant for both the SSE-Factor (A = 41.7%) and SSE-Single (A = 53.4%). Shared environmental effects were significant for the SSE-Factor (C = 28.9%), but did not reach significance for the SSE-Single (C = 17.4%; see Table 1). Unshared environmental effects explained the remaining 29% in both univariate models and are always retained in a univariate design as E incorporates measurement error.

**Table 1:** Univariate ACE estimates of objective and Self-evaluated singing ability (n_pair_ = 453)

	ACE Components [95% CI]			Intraclass Twin Correlations [95% CI]	
	A (%)	C (%)	E (%)	MZ	DZ
SPI	35.23*** [16.50, 59.32]	41.88*** [18.20, 59.80]	22.88*** [19.11, 27.37]	.77 [.72, .81]	.60 [.47, .71]
SSE-Factor	41.71*** [18.95, 69.66]	28.92* [1.73, 50.39]	29.37*** [24.63, 34.99]	.70 [.64, .75]	.52 [.37, .64]
SSE-Single	53.42*** [28.71, 74.72]	17.38 [0.00, 41.78]	29.20***	.71 [.65,.76]	.44 [.28,.57]

*n*_*pair*_reflects the total pair of twins (MZ and DZ) with complete data. *SPI* = singing phenotypic index, *SSE-Factor *= singing self-evaluation factor, *SSE-Factor *= singing self-evaluation single item, *CI* = confidence interval, *A* = additive genetic effects, *C* = shared environmental effects, *E* = unshared environmental effects. *E* is always retained in a univariate design as it incorporates measurement error.

* *p* <.05, **p <.01, ***p <.001

### Multivariate Analyses of Singing Self-evaluation

**Fig. 3:**
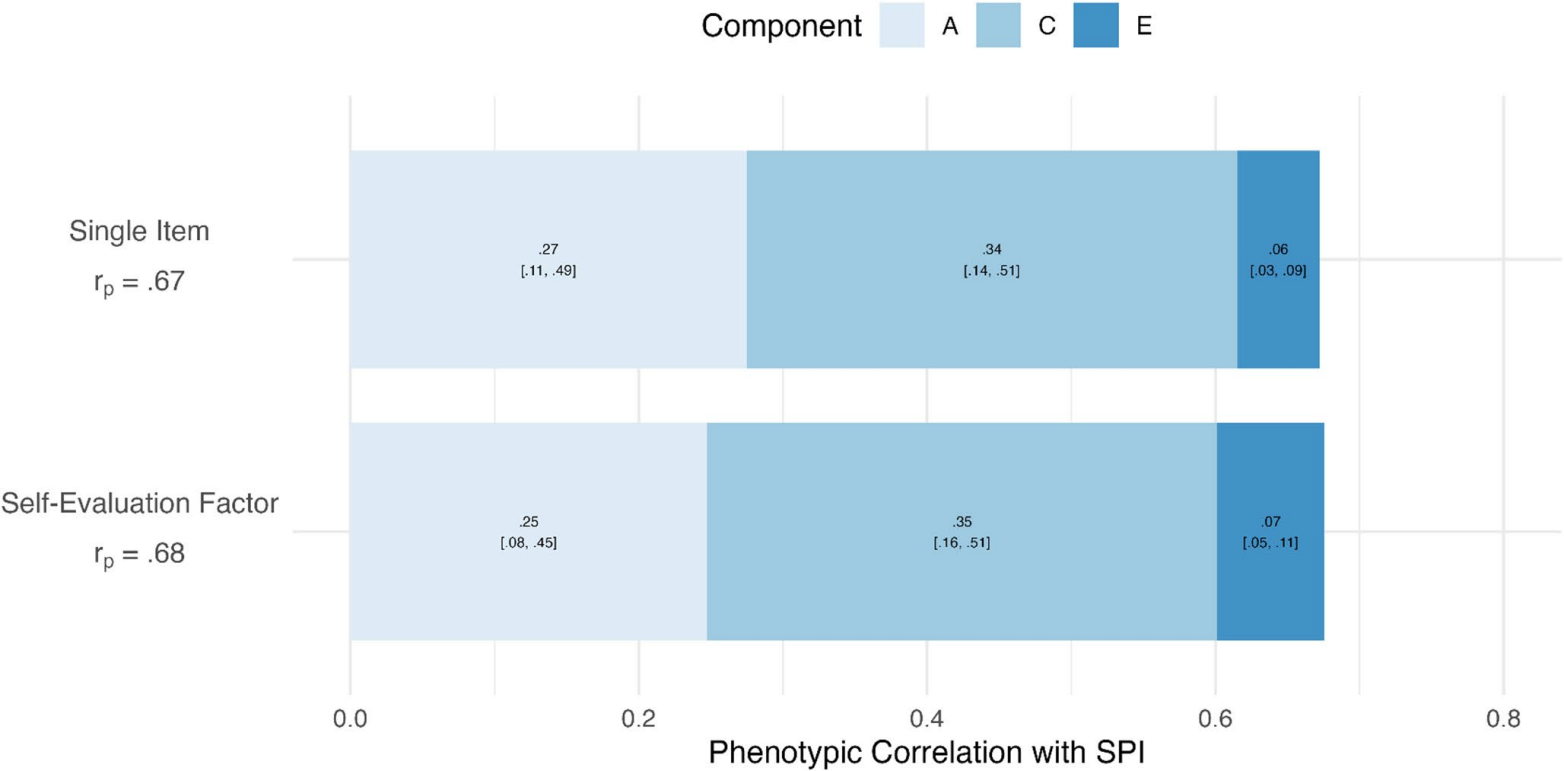
ACE contribution to phenotypic correlations between singing self-evaluation measures and objective ability (*n*_*pair*_ = 453). *n*_*pair*_ reflects the total pair of twins (MZ and DZ) with complete data. *SPI* = singing phenotypic index. *A* = additive genetic effects, *C* = shared environment effects, *E* = unshared environmental effects, *r*_*p*_ = total phenotypic correlation. The ACE estimates contribute additively to the phenotypic correlation. Numbers in brackets represent 95% confidence intervals.

Model fitting statistics for the multivariate model is presented in Table 2. All comparisons between the baseline ACE model and nested submodels were significant, indicating the full ACE model provided the best fit to the data. Table 3 presents the multivariate model estimates and the genetic and environmental correlations. Phenotypic correlations and respective ACE contributions (with 95% confidence intervals) are presented in Fig. 3, which illustrates the degree to which genetic, shared environmental, and unshared environmental factors contribute to the correlations. Variance and covariance estimates are given in Figure S1.

**Table 2:** Model fit statistics for multivariate analyses between objective and Self-Evaluated singing ability (*n*_*pair*_ = 453)

Model	EP	−2LL	df	AIC	Δ −2LL	Δ df	*p*
**ACE**	**30**	**6899.72**	**2688**	**6959.72**			
AE	24	6912.96	2694	6960.96	13.24	6	0.039**
CE	24	6941.98	2694	6989.98	42.26	6	< 0.001***
E	18	7451.58	2700	7487.58	551.86	12	< 0.001***

Each submodel is compared to the full model. *n*_*pair*_ reflects the total pair of twins (MZ and DZ) with complete data. *SPI* = singing phenotypic index, *SSE-Factor* = singing self-evaluation factor, *SSE-Single* = singing self-evaluation single item, *EP* = estimated parameters, *−2LL* = minus two times the log-likelihood, *df* = degrees of freedom, *AIC* = Akaike Information Criterion, Δ = change between baseline and comparison model. Best fitting model is reflected in bold

* *p* <.05, **p <.01, ***p <.001

Phenotypic correlations for the SSE-Factor (*r*_*p*_ = 0.68) and SSE-Single (*r*_*p*_ = 0.67) with the SPI were similar. Both phenotypic correlations were primarily explained by additive genetic (A = 36.6% and 40.9%) and shared environmental influences (C = 52.4% and 50.7%), with the remainder explained by unshared environmental influences (E = 11.0% and 8.4%) for the SSE-Factor and SSE-Single, respectively. The genetic and shared environmental correlations between the SPI and both the SSE-Factor (*r*_*g*_ = 0.62) and SSE-Single (*r*_*g*_ = 0.63) were substantial, indicating overlapping genetic factors between the phenotypes. Unshared environmental correlations were lower in comparison (*r*_*e*_ = 0.29 and 0.22).

Shared environmental correlations were estimated to be greater than 1, which can occur under direct symmetric models (Verhulst et al. 2019). To more robustly test the significance of these effects, we first fit three submodels where the C component on each variable (SPI, SSE-Factor, SSE-Single) was dropped by fixing their variance and covariances with other variables to zero. These reduced submodels were then compared to the full ACE model via likelihood ratio tests. In all three instances, dropping C led to significantly worse fit compared to the full ACE model (all *p*s < 0.05). Next, we constrained the shared environmental correlations to *r*_*c* =_ 0.90 to examine whether the data were compatible with such high shared environmental correlations. This constrained model was not a significantly worse fit than the original ACE model (Δ −2LL = 2.50, Δ *df* = 3, *p* = .475), suggesting that high shared environmental correlations between the variables were supported. Finally, to examine whether the estimates for C were similar for both self-evaluation measures, we fit another submodel that equated the free C parameters on both variables. This model also did not lead to significantly worse model fit (Δ −2LL = 0.724, Δ  = 1, *p* = .395), indicating there were no substantial differences in C influences between the variables.

These patterns of results did not change when factoring in years of music training as an additional covariate in the multivariate model (Table S3). The two self-evaluation measures were also highly genetically and environmentally correlated (*r*_*g*_ = 0.82, *r*_*c*_ = 0.96, *r*_*e*_ = 0.36), indicating that they also tapped into shared genetic and shared environmental factors. For readers interested in the individual SSE-Factor items, we present bivariate analyses between each item and the SPI in Tables S4 and S5.

**Table 3:** Multivariate ACE estimates between objective and Self-evaluated singing ability (*n*_*pair*_ = 453)

			% of r_p_			Genetic and Environmental Correlations [95% CI]			CTCT Correlations [95% CI]	
	r_p_		A (%)	C (%)	E (%)	r_g_	r_c_	r_e_	MZ	DZ
SSE-Factor and SPI	.68 [.63, .72]		36.64	52.35	11.00	.63 [.27, .92]	1^a^	.29 [.19, .38]	.60 [.55, .65]	.48 [.37, .56]
SSE-Single and SPI	.67 [.63, .71]		40.88	50.68	8.44	.64 [.31, .91]	1	.22 [.12, .32]	.62 [.56, .66]	.48 [.37, .57]

All estimates are derived from the multivariate correlated factors model.* n*_*pair*_reflects the total pair of twins (MZ and DZ) with complete data. *SPI* = singing phenotypic index,* SSE-Factor *= singing self-evaluation factor, *SSE-Single *= singing self-evaluation single item, *A* = additive genetic effects, *C* = shared environmental effects, *E* = unshared environmental effects, *CI *= confidence interval, *CTCT* = cross-twin cross-trait [correlations], *r*_*p*_ = total phenotypic correlation, *r*_*g*_ = genetic correlation, *r*_*c*_ = shared environmental correlation, *r*_e_ = unshared environmental correlation.

^a^ The value here exceeds 1, which can occur in direct symmetric parameterisations of twin models. For reporting purposes we report these as 1.

## Discussion

We showed that singing self-evaluation was significantly influenced by genetic and unshared environmental factors, using two minimal phenotypic measures: a composite self-evaluation factor (SSE-Factor), and a single item measure (SSE-Single). Shared environmental influences were only significant for the factor measure. Both self-evaluation measures also showed strong genetic and environmental correlations with objective ability, and their phenotypic correlations were largely explained by both genetic and shared environmental influences. Taken together, these results suggest that genes and the environment are related to both how individuals perceive their own singing ability, and the way these evaluations relate to their actual singing ability.

We initially predicted similar degrees of additive genetic and shared environmental effects on both singing self-evaluation measures, consistent with our findings on objective singing ability (Yeom et al. 2022). However, our univariate results only partially supported this hypothesis. Although both measures were influenced by additive genetic and unshared environmental effects (like other traits; Polderman et al. 2015), only the SSE-Factor measure was influenced by shared environments. We previously suggested that although the two self-evaluation measures predict objective ability to the same extent, people may rely on different sources of self-evaluative information when responding to factorial versus single-item measures (Yeom et al. 2023). Broadly speaking, accurate singing requires the development of an internal sensorimotor vocal model, which is largely acquired through experience (Tsang et al. 2011). The behaviours captured by the specific items in the SSE-Factor may reflect the extent to which this internal sensorimotor model has been developed, though this interpretation remains speculative as these processes were not directly measured. In contrast, the SSE-Single captures a more global and less nuanced evaluation of overall singing ability and has lower phenotypic resolution. Our findings show that it is more strongly influenced by individual experiences instead of experiences shared with a twin or family, likely relating to more general self-concepts of singing.

Our multivariate model showed that genetic and shared environmental factors predominantly explained the phenotypic correlations between self-evaluated and objective singing ability, while unshared environmental factors contributed less. The genetic correlations between both self-evaluation measures and objective ability were strong, indicating that singing self-evaluation likely shares genes with objective performance. This finding is consistent with the generalist genes hypothesis, which proposes the existence of shared genes that influence multiple cognitive abilities (Plomin et al. 2007). These generalist genes may operate at different levels of the learning process by influencing common cognitive abilities relevant for learning of more specialised abilities, including singing.

### Potential Mechanisms of Self-evaluation of Singing

**Fig. 4:**
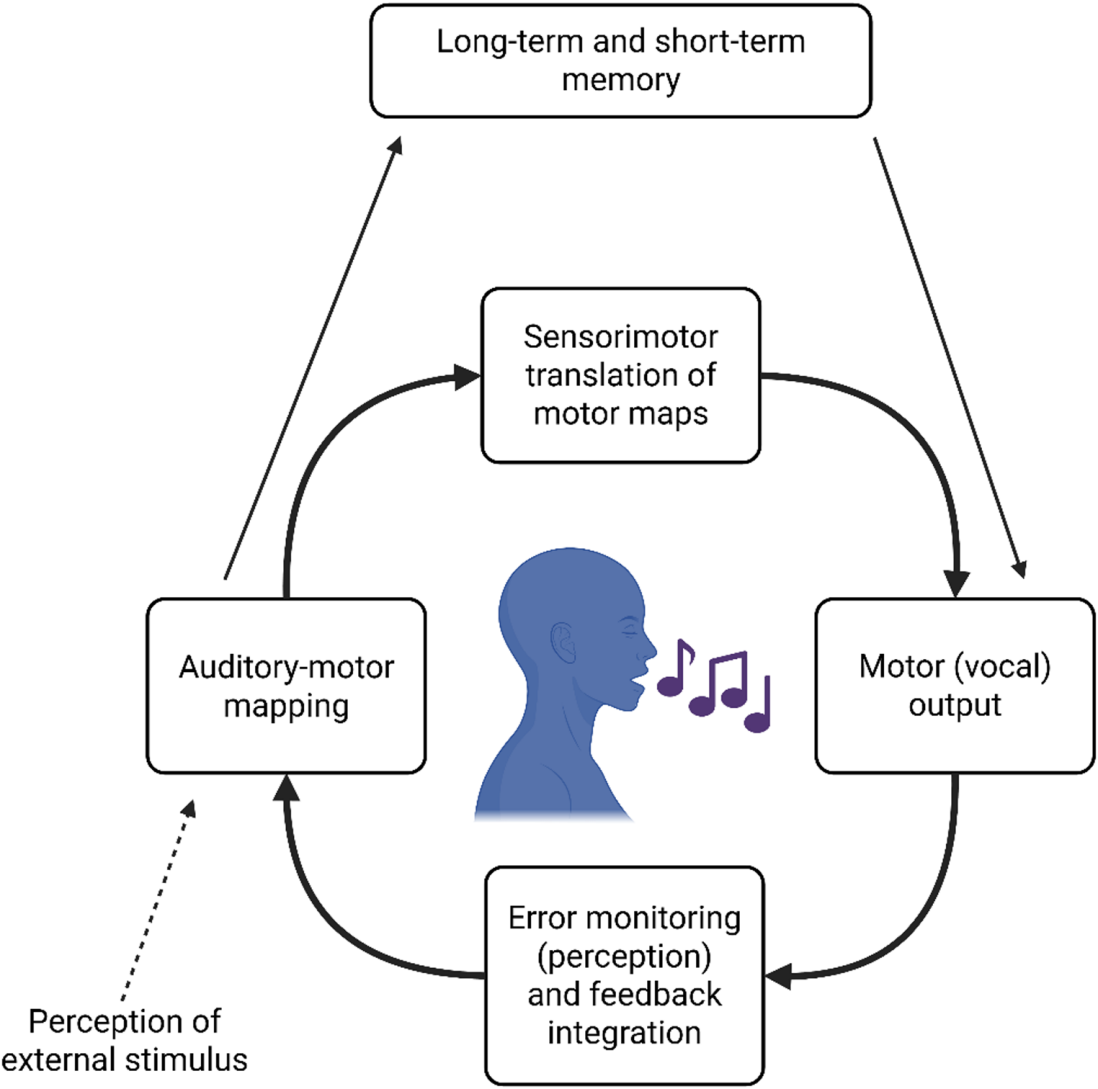
Schematic diagram of the Vocal Sensorimotor Loop. Adapted from Halpern and Pfordresher (2022). Graphic created in BioRender.

Although our results do not directly reflect the mechanisms that might explain these shared genetic bases, the Vocal Sensorimotor Loop (VSL; Berkowska and Dalla Bella 2009) provides a useful framework for unpacking what may underlie our observed effects. As shown in Fig. 4, the VSL is instrumental to singing, integrating memory, perception, auditory-motor mapping and motor planning (Berkowska and Dalla Bella 2009; see also Tsang et al. 2011). Based on this model, we speculate that these shared genes may be involved in sensorimotor processes. In the VSL, singing relies on immediate auditory feedback through which people self-monitor and adjust their vocal output (Berkowska and Dalla Bella 2009). This self-monitoring during singing primarily relies on sensorimotor processes to translate auditory-motor maps into subsequent motor plans, which are then executed as adjustments to the sung melody (Berkowska and Dalla Bella 2009; Greenspon and Montanaro 2023). A related mechanism may be interoception, the process of perceiving internal bodily signals. Singing is heavily dependent on interoceptive signals, such as the sensations produced by vibrating the vocal folds during singing (Zamorano et al. 2025). Higher interoceptive abilities are associated with singing accuracy independent of training (Zamorano et al. 2025). Interoception is functionally subserved by the anterior insula, which also guides vocal motor production, feedback monitoring and error correction during singing (Kleber et al. 2010, 2013; Zamorano et al. 2025). Being aware of internal signals during singing, and the immediacy of external auditory feedback, may foster both accurate singing and an embodied awareness of singing accuracy (Zamorano et al. 2025). These mechanisms may also help explain why self-evaluations of singing correlate strongly with objective performance. In short, the shared genetic bases observed between self-evaluated and objective singing ability may primarily reflect sensorimotor and interoceptive processes.

We note that given the relatively modest sample size, we may have been somewhat underpowered to detect robust and stable estimates of the shared environment. Our extremely high shared environmental correlation estimates (*r* ~ 1) may be boundary effects that reflect this model instability. However, given the significant univariate shared environment estimate for the SSE-Factor, and the better fit of the full multivariate ACE model, we suggest that shared environmental effects may still be relevant for the relationship between self-evaluated and objective singing ability. Sensorimotor processes are also shaped by environmental factors, which may explain the substantial shared environmental correlations between the self-evaluative and objective measures. Early familial environments may be especially formative for developing singing ability, in part due to the possibility of a sensitive period for singing in childhood (Yeom et al. 2022, 2024). During this sensitive period, an internal sensorimotor model for singing may develop most rapidly (Yeom et al. 2024). This aligns with prior evidence suggesting a sensitive period for general sensorimotor processes (Penhune 2020; Tsang et al. 2011). Frequent singing engagement during early life may therefore support fine-tuning of the sensorimotor processes underlying singing ability, including the learning mechanisms of the VSL, through regular opportunities for feedback. The refinement of these sensorimotor processes will also subsequently shape self-evaluations of singing ability.

Musically enriched familial environments may also provide frequent opportunities for individuals to receive external feedback through regular exposure and engagement with singing, contributing to the formation of self-concepts and self-evaluations of singing (Leary 2007; Sedikides and Strube 1997; Taylor et al. 1995). Family members may provide direct feedback on an individual’s singing accuracy, which can be crucial for forming people’s self-concepts around how well they believe they can sing (Whidden 2010). Additionally, consistent exposure to family members participating in singing activities may also facilitate comparative assessments of ability between the individual and others. Finally, individuals with a genetic predisposition for singing ability might also inherit or self-select environments that offer more opportunities for singing engagement – in turn, contributing to opportunities for feedback within this environment. This would be indicative of gene-environmental correlations (Plomin et al. 1977) that cannot be tested in the present study. Regardless, it is possible that familial feedback within a shared environment may shape individuals’ attitudes towards their own singing ability and impact their participation in singing activities either in a positive or negative way. Future work with larger sample sizes would be valuable for obtaining more stable estimates of shared environmental effects.

We note that the observed difference in shared environmental effects between the univariate and multivariate analyses for the SSE-Single are most likely explained by our choice to use both univariate and multivariate analyses. Multivariate analyses capture the covariation between two traits, allowing for detection of influences that might not be apparent when examining univariate traits in isolation (Rijsdijk and Sham 2002). Multivariate twin models also have greater power to detect univariate influences on traits, and thus may provide more precise estimates of shared environmental effects (Schmitz et al. 1998). Our findings indicate that shared environmental effects could be equated between the SSE-Factor and SSE-Single in our multivariate model, suggesting that both SSE measures may be similarly influenced by shared environment. As noted above, future work would be useful for clarifying these effects further. Finally, the contribution of shared environmental influences to a phenotypic relationship is a function of both univariate shared environmental influences and their shared environmental correlation, as is the case for the contribution of genetic effects. The significant shared environmental influences on objective singing ability (Yeom et al. 2022) and the high overlap in environmental factors with self-evaluation are thus likely to explain the significant contribution of shared environment to the phenotypic relationship in the SSE-Single despite a non-significant C in the univariate model. In short, shared environmental influences on singing ability also largely influence self-evaluations, whereas specific shared environmental influences are small.

Our findings support the validity of using either the SSE-Factor or SSE-Single as minimal phenotypic measures for investigating singing ability (Yeom et al. 2023), and suggest they may be viable measures for future genome-wide association studies (GWAS) of singing. Both measures have higher phenotypic correlations than the individual items that make up the SSE-Factor and share substantial genetic correlations with objective singing ability. Here, it is important to note that the SSE-Single measure will have lower phenotypic resolution than the SSE-Factor by design, which captures a range of specific singing behaviours. Existing GWAS efforts comparing multi-item versus single item measures have shown that multi-item measures may capture more SNP-based heritability (Jamshidi et al. 2020). However, whether this would be the case for our SSE-Factor and SSE-Single measures would need to be empirically tested. Despite this, the high phenotypic and genetic correlations between singing self-evaluation and singing ability suggest that these phenotypes may share underlying genes. Thus, our self-report measures accurately capture singing ability and may also capture genetic loci relevant for singing. Given the recent advent of large-scale GWAS for musicality (Niarchou et al. 2022), we propose that both measures presented here can serve as viable and suitable minimal phenotypic measures of singing ability.

### Limitations

There are some limitations in our study. Some of the limitations outlined in our previous studies (Yeom et al. 2022, 2023) are relevant here. This includes the high proportion of women in our sample, which reflects the composition of the Twins Research Australia database (Murphy et al. 2019) and may limit generalisability. Self-selection bias may also have occurred, as individuals with a greater interest in music or singing may have been more likely to participate. Additionally, because data collection occurred online, participation required internet access and some familiarity with technology, which may have introduced further selection bias. Participants also completed the questionnaire items directly after the singing tasks, potentially aiding in the accuracy of the self-evaluations; however, order effects tend to be modest at best (Zell and Krizan 2014). The cross-sectional nature of this study limits inferences about the relationship between singing self-evaluation and singing ability in terms of its directionality and change over time. Future research using a cross-lagged design, similar to Luo et al. (2011), may shed light on the exact nature on the bidirectional relationship between self-evaluation and objective singing. A related question worth investigating is whether genetic and environmental effects on self-evaluation also differ throughout life. Finally, our sample size of 453 complete twin pairs was modest, which may explain the instability in the estimates of shared environmental effects. Future investigations in larger samples would allow for more precise model estimates.

## Conclusion

Our study is the first to investigate the genetic and environmental influences on singing self-evaluation and its relationship to singing ability. Both singing self-evaluation and its relationship with objective singing ability are influenced by shared genetic and environmental factors. These shared genetic and environmental factors point to potential mechanisms related to sensorimotor integration, a skill that may be refined during a sensitive period early in life. We also demonstrate genetically informative validation for two measures of singing self-evaluation from our previous work (Yeom et al. 2023), which may serve as suitable minimal phenotypes for future gene discovery. Broadly, our findings point to the complexity of factors that shape not only how our skills and abilities develop, but also how we understand and self-evaluate these abilities.

## Supplementary Information

Below is the link to the electronic supplementary material.

Supplementary Material 1

## Data Availability

The data for this study cannot be shared publicly, as ethical approval to openly share data was not sought at the time the study began. Therefore, the data are subject to restrictions by both our institutional review board and Twins Research Australia. Requests for the data should be directed to the corresponding author.
