# Supplementary material for: Genetic and Environmental Influences on Singing Self-Evaluation and its Relationship with Singing Ability: An Australian Twin Study
Source: Behav Genet. 2026 Feb 9;56(2):66–79. doi: 10.1007/s10519-026-10254-4 (PMC13132946; doi:10.1007/s10519-026-10254-4)
Supplement: Supplementary file 1 — Supplementary Material 1 [file 10519_2026_10254_MOESM1_ESM.docx]

**Genetic and environmental influences on singing self-evaluation and its relationship with singing ability: An Australian twin study**

**Supplementary Information**

Daniel Yeom ^1^ *, Kendall S. Stead ^1^ *, Yi Ting Tan ^2^, Gary E. McPherson ^2^, Miriam A. Mosing ^1 3 4^, Sarah J. Wilson ^1 5^

**ORCID IDs:**

Daniel Yeom: <https://orcid.org/0000-0002-1281-6299>

Kendall Stead: <https://orcid.org/0000-0001-9645-2439>

Gary E. McPherson: <https://orcid.org/0000-0002-2543-6762>

Miriam A. Mosing: <https://orcid.org/0000-0003-2049-125X>

Sarah J. Wilson: <https://orcid.org/0000-0002-2678-1576>

**Figure S1**. Variance decomposition of the correlated factors model between the Singing Phenotypic Index, the singing self-evaluation factor (SSE-Factor) and the single self-evaluation item (SSE-Single).


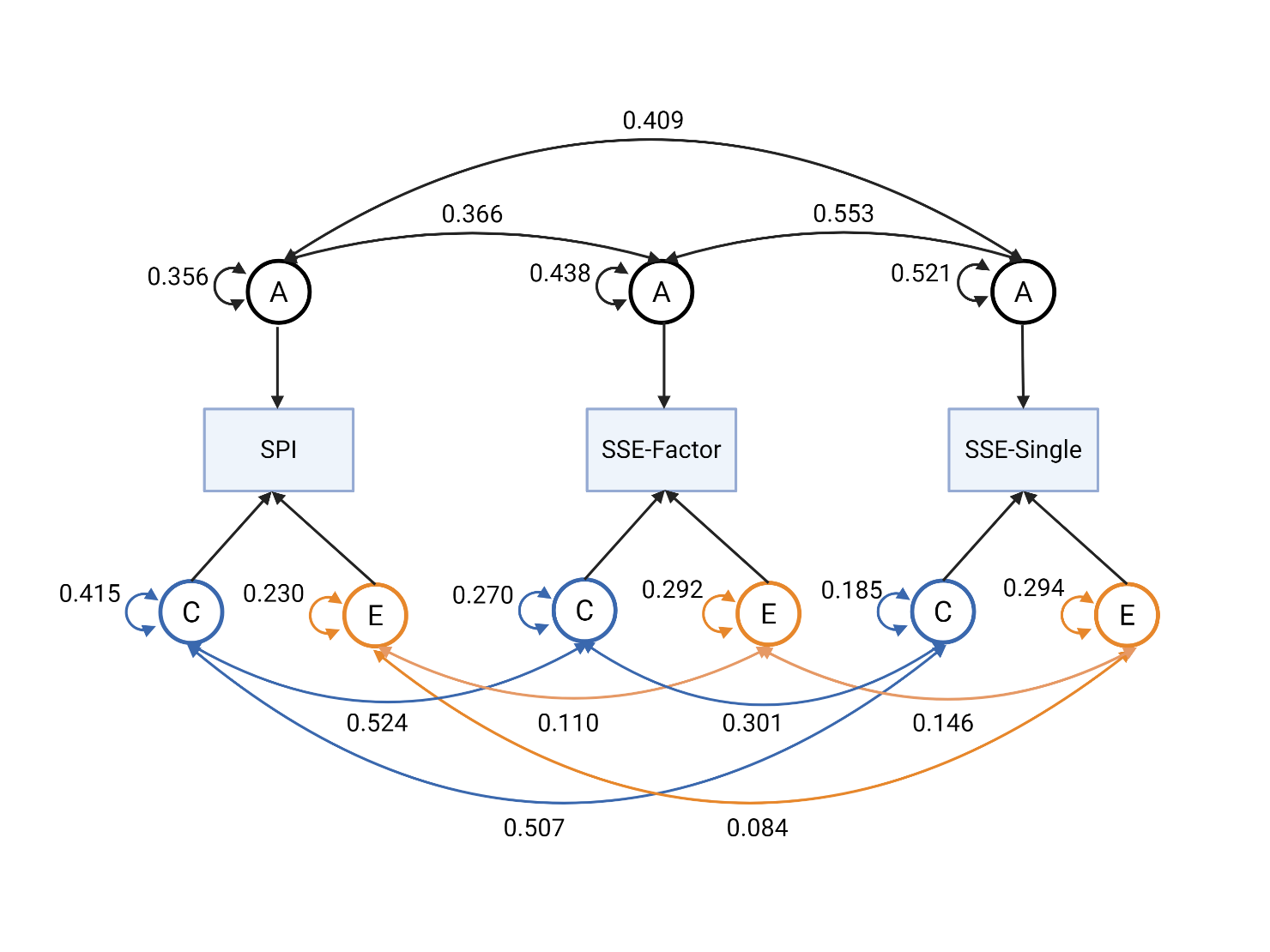


*Note.* Standardised variances are displayed. For visualisation purposes, the C components have been coloured blue and the E components have been coloured orange. SPI = Singing Phenotypic Index; SSE-Factor = Singing Self-Evaluation Factor; SSE-Single = Singing Self-Evaluation Single item.

**Table S1.** *Assumption Testing Statistics for Genetic Analyses (n_pair_* = 453)

| Variable | Base | Comparison | ep | -2LL | df | AIC | Δ -2LL | Δ df | *p* |
| --- | --- | --- | --- | --- | --- | --- | --- | --- | --- |
| SPI | Saturated |  | 27 | 2164.83 | 879 | 2218.83 |  |  |  |
|  | Saturated | Equal Means Twin Order | 23 | 2167.44 | 883 | 2213.44 | 2.61 | 4 | 0.625 |
|  | Equal Means Twin Order | Equal Means SS Zygosity | 21 | 2168.96 | 885 | 2210.96 | 1.51 | 2 | 0.470 |
|  | Equal Means SS Zygosity | Equal Means All Zygosity | 19 | 2176.45 | 887 | 2214.45 | 7.50 | 2 | 0.024 |
|  | Equal Means All Zygosity | Equal Means | 18 | 2176.45 | 888 | 2212.45 | -0.00 | 1 | 1 |
|  | Equal Means | Equal Variance Twin | 14 | 2176.98 | 892 | 2204.98 | 0.53 | 4 | 0.970 |
|  | Equal Variance Twin | Equal Variance SS Zygosity | 12 | 2177.15 | 894 | 2201.15 | 0.17 | 2 | 0.918 |
|  | Equal Variance SS Zygosity | Equal Variance All Zygosity | 10 | 2177.18 | 896 | 2197.18 | 0.02 | 2 | 0.988 |
|  | Equal Variance All Zygosity | Equal Variance | 9 | 2178.65 | 897 | 2196.65 | 1.47 | 1 | 0.225 |
|  | Equal Variance | Equal Covariance MZ | 8 | 2178.67 | 898 | 2194.67 | 0.02 | 1 | 0.886 |
|  | Equal Covariance MZ | Equal Covariance DZ | 7 | 2178.67 | 899 | 2192.67 | 0.01 | 1 | 0.936 |
|  | Equal Covariance DZ | Equal Covariance MZ DZ All | 6 | 2178.68 | 900 | 2190.68 | 0.00 | 1 | 0.976 |
| Self-Evaluation | Saturated |  | 27 | 2288.76 | 879 | 2342.76 |  |  |  |
|  | Saturated | Equal Means Twin Order | 23 | 2290.47 | 883 | 2336.47 | 1.70 | 4 | 0.790 |
|  | Equal Means Twin Order | Equal Means SS Zygosity | 21 | 2290.65 | 885 | 2332.65 | 0.18 | 2 | 0.914 |
|  | Equal Means SS Zygosity | Equal Means All Zygosity | 19 | 2295.57 | 887 | 2333.57 | 4.93 | 2 | 0.085 |
|  | Equal Means All Zygosity | Equal Means | 18 | 2295.57 | 888 | 2331.57 | -0.00 | 1 | 1 |
|  | Equal Means | Equal Variance Twin Order | 14 | 2296.02 | 892 | 2324.02 | 0.45 | 4 | 0.978 |
|  | Equal Variance Twin Order | Equal Variance SS Zygosity | 12 | 2297.07 | 894 | 2321.07 | 1.05 | 2 | 0.593 |
|  | Equal Variance SS Zygosity | Equal Variance All Zygosity | 10 | 2297.09 | 896 | 2317.09 | 0.02 | 2 | 0.992 |
|  | Equal Variance All Zygosity | Equal Variance | 9 | 2297.49 | 897 | 2315.49 | 0.40 | 1 | 0.527 |
|  | Equal Variance | Equal Covariance MZ | 8 | 2302.73 | 898 | 2318.73 | 5.25 | 1 | 0.022 |
|  | Equal Covariance MZ | Equal Covariance DZ | 7 | 2302.81 | 899 | 2316.81 | 0.07 | 1 | 0.786 |
|  | Equal Covariance DZ | Equal Covariance MZ DZ All | 6 | 2303.20 | 900 | 2315.20 | 0.39 | 1 | 0.532 |
| Single Item | Saturated |  | 27 | 2292.73 | 879 | 2346.73 |  |  |  |
|  | Saturated | Equal Means Twin Order | 23 | 2297.49 | 883 | 2343.49 | 4.75 | 4 | 0.313 |
|  | Equal Means Twin Order | Equal Means SS Zygosity | 21 | 2297.60 | 885 | 2339.60 | 0.11 | 2 | 0.946 |
|  | Equal Means SS Zygosity | Equal Means All Zygosity | 19 | 2298.09 | 887 | 2336.09 | 0.49 | 2 | 0.781 |
|  | Equal Means All Zygosity | Equal Means | 18 | 2298.09 | 888 | 2334.09 | 0.00 | 1 | 1 |
|  | Equal Means | Equal Variance Twin Order | 14 | 2299.68 | 892 | 2327.68 | 1.59 | 4 | 0.810 |
|  | Equal Variance Twin Order | Equal Variance SS Zygosity | 12 | 2299.77 | 894 | 2323.77 | 0.08 | 2 | 0.960 |
|  | Equal Variance SS Zygosity | Equal Variance All Zygosity | 10 | 2300.32 | 896 | 2320.32 | 0.55 | 2 | 0.760 |
|  | Equal Variance All Zygosity | Equal Variance | 9 | 2300.32 | 897 | 2318.32 | 0.00 | 1 | 0.964 |
|  | Equal Variance | Equal Covariance MZ | 8 | 2300.33 | 898 | 2316.33 | 0.01 | 1 | 0.924 |
|  | Equal Covariance MZ | Equal Covariance DZ | 7 | 2301.47 | 899 | 2315.47 | 1.14 | 1 | 0.286 |
|  | Equal Covariance DZ | Equal Covariance MZ DZ All | 6 | 2301.77 | 900 | 2313.77 | 0.31 | 1 | 0.580 |

*Note.* *n_pair_* reflects the total pair of twins (MZ and DZ) with complete data. Level of significance for each comparison is *p* < .01. *SPI* = singing phenotypic index. *Base* = baseline model, *ep* = estimated parameters, *-2LL* = minus two times the log-likelihood, *df* = degrees of freedom, *AIC* = Akaike Information Criterion, Δ = change between baseline and comparison model.

**Table S2.** *Model Fitting Statistics for Univariate Genetic Analyses (n_pair_* = 453).

| Variable | Base | Comparison | ep | -2LL | df | AIC | Δ -2LL | Δ df | *p* |
| --- | --- | --- | --- | --- | --- | --- | --- | --- | --- |
| SPI | ACE |  | 6 | 2178.68 | 900 | 2190.68 |  |  |  |
|  | ACE | AE | 5 | 2188.83 | 901 | 2198.83 | 10.16 | 1 | <.001*** |
|  | ACE | CE | 5 | 2194.73 | 901 | 2204.73 | 16.05 | 1 | <.001*** |
|  | ACE | E | 4 | 2531.44 | 902 | 2539.44 | 352.77 | 2 | <.001*** |
| Self-Evaluation Factor | ACE |  | 6 | 2303.20 | 900 | 2315.20 |  |  |  |
|  | ACE | AE | 5 | 2307.49 | 901 | 2317.49 | 4.29 | 1 | .038* |
|  | ACE | CE | 5 | 2317.95 | 901 | 2327.95 | 14.75 | 1 | <.001*** |
|  | ACE | E | 4 | 2564.79 | 902 | 2572.79 | 261.59 | 2 | <.001*** |
| Single Item | ACE |  | 6 | 2301.77 | 900 | 2313.77 |  |  |  |
|  | ACE | AE | 5 | 2303.11 | 901 | 2313.11 | 1.34 | 1 | .247 |
|  | ACE | CE | 5 | 2322.99 | 901 | 2332.99 | 21.22 | 1 | <.001*** |
|  | ACE | E | 4 | 2561.12 | 902 | 2569.12 | 259.34 | 2 | <.001*** |

*Note.* *n_pair_* reflects the total pair of twins (MZ and DZ) with complete data. *SPI* = singing phenotypic index. *Base* = baseline model, *ep* = estimated parameters, *-2LL* = minus two times the log-likelihood, *df* = degrees of freedom, *AIC* = Akaike Information Criterion, Δ = change between baseline and comparison model.

**p* < .05, ***p* < .01, ****p* < .001

**Table S3.** *ACE Estimates Between Objective and Self-Evaluated Singing Ability, Controlling for Years of Music Training (n_pair_* = 450).

|  |  | | % of r_p_ | | |  | Genetic and Environmental Correlations  [95% CI] | | |  | CTCT Correlations  [95% CI] | |
| --- | --- | --- | --- | --- | --- | --- | --- | --- | --- | --- | --- | --- |
|  | r_p_ | A (%) | | C (%) | E (%) |  | r_g_ | r_c_ | r_e_ |  | MZ | DZ |
| SSE-Factor and SPI | .63  [.58, .67] | 38.49 | | 48.25 | 13.26 |  | .62  [.21, .98] | 1 | .28  [.28, .37] |  | .54  [.48, .60] | .41  [.31, .51] |
| SSE-Single and SPI | .62  [.57, .67] | 44.17 | | 44.98 | 10.86 |  | .62  [.25, .93] | 1^a^ | .22  [.11, .32] |  | .55  [.49, .60] | .41  [.30, .51] |

*Note.* All estimates are derived from the multivariate correlated factors model. Years of training was winsorised to a maximum of 20 years to account for implausible values. *n_pair_* reflects the total pair of twins (MZ and DZ) with complete data. *SPI* = singing phenotypic index, *SSE-Factor* = singing self-evaluation factor, *SSE-Factor* = singing self-evaluation single item, *CI* = confidence interval, *CTCT* = cross-twin cross-trait [correlations], *r_p_* = total phenotypic correlation, *r_g_* = genetic correlation, *r_c_* = shared environmental correlation, *r_e_* = unshared environmental correlation.

^a^ The value here exceeds 1, which can occur in direct symmetric parameterisations of twin models. For reporting purposes we report these as 1.

**Bivariate analyses of individual items in the SSE-Factor.** The SSE-Factor is made up of four self-report items related to singing. We fit bivariate Cholesky models to examine the genetic and environmental correlations/relationships between each item and the Singing Phenotypic Index. These are presented in Tables S4 and S5 below.

**Table S4.** *Bivariate ACE estimates Between Individual Singing Self-Evaluation Factor Items with Objective Singing Ability (n_pair_* = 453)

|  |  | % of *r_p_* | | |  | | Genetic and environmental correlations [95% CI] | | |
| --- | --- | --- | --- | --- | --- | --- | --- | --- | --- |
| Variable | r_p_ | A (%) | C (%) | E (%) | |  | r_g_ | r_c_ | r_e_ |
| *I can tell when people sing out of tune.* | .57 | 57.01* | 33.61 | 9.38* | |  | .74*  [.74, 1] | .91  [-1, 1] | .18*  [.08, .28] |
| *I find it easy to sing, hum or whistle a familiar tune from memory.* | .51 | 52.08* | 39.65* | 8.28* | |  | .78*  [.34, 1] | .60*  [.60, 1] | .14*  [.03, .24] |
| *After hearing a new song two or three times, I can usually sing/hum/whistle it by myself.* | .48 | 24.61 | 62.33* | 13.06* | |  | .40  [-1, .69] | 1*  [.78, 1] | .18*  [.07, .28] |
| *When I sing/hum/whistle, I have no idea whether I’m in tune or not. ^a^* | .54 | 51.42* | 34.76* | 13.82* | |  | .74*  [.74, 1] | 1*  [.95, 1] | .21*  [.11, .31] |

*Note. n_pair_* reflects the total pair of twins (MZ and DZ) with complete data. *SPI* = singing phenotypic index, *A* = additive genetic effects, *C* = shared environmental effects, *E* = unshared environmental effects, *CI* = confidence interval, *r_p_* = total phenotypic correlation, *r_g_* = genetic correlation, *r_c_* = shared environmental correlation, *r_e_* = unshared environmental correlation.

^a^ This item was reverse-scored.

* *p* <.05

**Table S5.** *Model Fit Statistics for Bivariate Analyses of Individual SSE-Factor Items (n_pair_* = 453).

| Variable | Comparison | EP | -2LL | df | AIC | Δ -2LL | Δ df | *p* |
| --- | --- | --- | --- | --- | --- | --- | --- | --- |
| *I can tell when people sing out of tune.* | Full model | 17 | 4493.26 | 1963 | 4527.26 |  |  |  |
|  | r_g_ = 0 | 16 | 4509.34 | 1964 | 4541.34 | 16.08 | 1 | < .001*** |
|  | **r_c_ = 0** | **16** | **4496.75** | **1964** | **4528.75** | **3.48** | **1** | **.06** |
|  | r_e_ = 0 | 16 | 4504.74 | 1964 | 4536.74 | 11.47 | 1 | < .001*** |
| *I find it easy to sing, hum or whistle a familiar tune from memory.* | Full model | 17 | 4820.75 | 1963 | 4854.75 |  |  |  |
|  | r_g_ = 0 | 16 | 4829.77 | 1964 | 4861.77 | 9.02 | 1 | < .001*** |
|  | **r_c_ = 0** | **16** | **4824.72** | **1964** | **4856.72** | **3.97** | **1** | **.05** |
|  | r_e_ = 0 | 16 | 4827.66 | 1964 | 4859.66 | 6.91 | 1 | .01** |
| *After hearing a new song two or three times, I can usually sing/hum/whistle it by myself.* | Full model | 17 | 4861.94 | 1963 | 4895.94 |  |  |  |
|  | **r_g_ = 0** | **16** | **4864.11** | **1964** | **4896.11** | **2.16** | **1** | **.14** |
|  | r_c_ = 0 | 16 | 4871.97 | 1964 | 4903.97 | 10.03 | 1 | < .001*** |
|  | r_e_ = 0 | 16 | 4873.34 | 1964 | 4905.34 | 11.40 | 1 | < .001*** |
| *When I sing/hum/whistle, I have no idea whether I’m in tune or not. ^a^* | **Full model** | **17** | **5237.71** | **1963** | **5271.71** |  |  |  |
|  | r_g_ = 0 | 16 | 5248.50 | 1964 | 5280.50 | 10.79 | 1 | < .001*** |
|  | r_c_ = 0 | 16 | 5242.31 | 1964 | 5274.31 | 4.60 | 1 | .03* |
|  | r_e_ = 0 | 16 | 5254.27 | 1964 | 5286.27 | 16.56 | 1 | < .001*** |
| Personal Engagement Factor | Full model | 17 | 4403.81 | 1795 | 4437.81 |  |  |  |
|  | **r_g_ = 0** | **16** | **4405.19** | **1796** | **4437.19** | **1.38** | **1** | **.24** |
|  | **r_c_ = 0** | **16** | **4407.13** | **1796** | **4439.13** | **3.32** | **1** | **.07** |
|  | r_e_ = 0 | 16 | 4408.16 | 1796 | 4440.16 | 4.35 | 1 | .04* |
| Social Engagement Factor | Full model | 17 | 4359.05 | 1795 | 4393.05 |  |  |  |
|  | **r_g_ = 0** | **16** | **4361.08** | **1796** | **4393.08** | **2.03** | **1** | **.15** |
|  | r_c_ = 0 | 16 | 4366.47 | 1796 | 4398.47 | 7.42 | 1 | .01** |
|  | r_e_ = 0 | 16 | 4368.43 | 1796 | 4400.43 | 9.39 | 1 | < .001*** |

*Note.* Each submodel is compared to the full model. *n_pair_* reflects the total pair of twins (MZ and DZ) with complete data. *EP* = estimated parameters, *-2LL* = minus two times the log-likelihood, *df* = degrees of freedom, *AIC* = Akaike Information Criterion, Δ = change between baseline and comparison model. Best fitting models are reflected in bold.

^a^ This item was reverse-scored.

* *p* < .05, ***p* < .01, ****p* < .001
